# Sex chromosome genes contribute to cocaine vulnerability in a strain-dependent manner

**DOI:** 10.21203/rs.3.rs-9519927/v1

**Published:** 2026-05-08

**Authors:** Aaron L. Le, Allison N. Dickey, Thanh-Binh Duong, Emilie F. Rissman, Wendy J. Lynch

**Affiliations:** University of Virginia; North Carolina State University; North Carolina State University; North Carolina State University; University of Virginia

**Keywords:** cocaine, Toll-like receptors, sex differences, estrogen, sex chromosomes, mouse strain differences

## Abstract

**Objective:**

We used wild-type C57BL/6J (B6) mice and four core genotype (FCG) mice on two strain backgrounds (B6 and MF1) to examine the role of SCC, estradiol, and genetic background in vulnerability to cocaine use.

**Results:**

In wild-type B6 mice, no effects of sex, gonadectomy, or estradiol treatment were observed during acquisition or progressive-ratio testing. In contrast, in gonadectomized B6 FCG mice, vulnerability was driven by SCC, with XY mice showing greater acquisition and responding than XX mice. SCC effects were not observed in MF1 FCG mice, indicating strain dependence. Whole-genome sequencing identified a missense single nucleotide polymorphism in *Zfy2*, consistent with Y-chromosome variation in the B6 FCG line. Anogenital distance was reduced in FCG B6 mice, suggesting altered early androgen exposure. Expression of toll-like receptor genes (*Tlr7, Tlr8*), and their association with cocaine self-administration behavior, differed by SCC and strain.

**Conclusion:**

SCC can influence cocaine vulnerability in a strain- and hormone-dependent manner; however, these effects are not generalizable across FCG models and may reflect interactions with strain-specific genetic features, including the X-to-Y chromosome translocation present in the B6 FCG line. Candidate mechanisms include Y-linked variation, developmental androgen exposure, and immune signaling; however, their functional relevance remains to be established.

## Introduction

Drug overdose deaths have been increasing at an alarming rate since 2019, with cocaine being the third leading cause (NIDA 2024). Women are more vulnerable than men to the rewarding effects of addictive drugs (Haas and Peters 2000; Chen and Kandel 2002; Sanvicente-Vieira et al. 2019). Rats have been used successfully to model this enhanced vulnerability in females and to investigate its underlying mechanisms (Jackson et al. 2006; Lynch 2008; Towers et al. 2023). These studies show that female rats acquire cocaine self-administration faster, and are more motivated to obtain infusions of cocaine than males (Lynch et al. 2001; Becker and Koob 2016; dos Anjos Rosário et al. 2022). They also identify estradiol as a critical contributor to these sex differences. However, work in this area has focused almost exclusively on estradiol, and other biologically relevant factors that may contribute to female vulnerability, including sex chromosome complement (SCC), remain largely unexplored (Martini et al. 2020; Le et al. 2023).

Mice offer superior genetic tools compared to rats, making them ideal for investigating contributing factors like SCC. Our group used the four core genotype (FCG) mouse model, which allows for an independent assessment of SCC (XX, XY) and gonadal sex (ovaries, testes), to examine mechanisms underlying sex differences in vulnerability to cocaine use (De Vries et al. 2002). These mice produce four genotypes: gonadal females with XX or XY chromosomes and gonadal males with XX or XY chromosomes (De Vries et al. 2002). When we tested gonadally-intact adult FCG mice on a C57BL/6J (B6) background (Martini et al. 2020), similar to findings in rats, we found that XY males were **less likely** to acquire cocaine self-administration than gonadal females of either SCC (XX or XY). This effect may reflect a more general reward difference, however, effects in gonadectomized FCG mice have generally been opposite to findings in rats and intact mice. For example, we found that XY male mice acquired cocaine self-administration more quickly than XX male mice and XY female mice, regardless of gonadal sex (Le et al. 2023). Similarly, another group showed that gonadectomy reversed the sex difference in food motivation in FCG mice, with XY mice of both gonadal sexes obtaining more food rewards under a progressive-ratio (PR) schedule than XX mice (Seu et al. 2014). Also in contrast to findings in rats, in the gonadectomized FCG mice, estradiol treatment failed to enhance acquisition in XX mice, and instead, reduced acquisition in XY males (Le et al., 2023). Together, these findings indicate that factors other than estradiol, including SCC, may contribute to vulnerability to cocaine use and may interact with gonadal hormones in complex ways, although differences in genetic background may also contribute. In particular, the FCG model, which was derived on a B6 background, is now known to carry a 3.2 MB region X-to-Y chromosome translocation (Panten et al. 2024). Thus, findings in B6 FCG mice may reflect SCC per se, interactions with gonadal hormones, or features of the B6 FCG genetic background.

To further investigate these possibilities, we explored interactions between gonadal hormones and SCC on vulnerability to cocaine as measured by acquisition under a fixed-ratio 1 (FR1) schedule and subsequent motivation under a PR schedule. Effects were examined in intact and gonadectomized wild-type (WT) B6 male and female mice, with and without estradiol. To evaluate potential contributions of sex SCC independent of genetic background, we used two strains implementing the FCG model: the original B6 line that harbors the X-to-Y chromosome translocation, and the MF1 line which does not carry the X-to-Y chromosome translocation (Panten et al. 2024). We also examined anogenital distance (AGD) in WT B6 mice and FCG B6 mice, an indirect marker of androgen exposure during development (McEwen et al. 1977; Kundakovic and Tickerhoof 2024; Lee et al. 2025), and assessed gene expression in the nucleus accumbens (NAc), with particular focus on Y-linked variation that could contribute to behavioral differences. Among the genes of interest, we honed in on two immune-related genes, toll-like receptors 7 and 8 (Tlr7 and Tlr8), which are duplicated on the FCG Y-chromosome and have been linked to addiction-related behaviors (Crews et al. 2017; Burkovetskaya et al. 2020; Montagud-Romero et al. 2021).

We hypothesized that gonadectomy and exogenous estradiol treatment would differentially affect vulnerability to cocaine use in male and female WT B6 mice. We further hypothesized that if SCC-related effects observed in FCG B6 persisted in the MF1 strain, then duplicated genes on the FCG B6 Y chromosome would be unlikely to account for the behavioral differences. Given the translocation difference between WT and FCG B6 lines, we also predicted differences in AGD between WT and FCG B6 mice.

Three experiments were conducted to test these hypotheses. In Experiment 1, we used B6 mice to evaluate the effects of the gonadectomy and estradiol treatment, addressing inconsistencies in prior findings in gonadally intact mice (Griffin et al. 2007; Engeln et al. 2020; López et al. 2021a). In Experiment 2, we examined the roles sex and SCC effects in gonadectomized B6 FCG mice, using a more gradual and lower-dose cocaine regime to better resolve group differences (Le et al. 2023). Lastly, in Experiment 3 we tested whether SCC-related effects observed in the B6 FCG line persist in MF1 FCG line, which lacks the X- to-Y-chromosome translocation.

## Methods

### Animals

All mice were generated in our colony (at NCSU). We bred original B6 mice (from Jackson Labs, Bar Harbor, ME, Stock #000664). We also purchased the FCG B6 XY^**−**^*Sry* breeder males from Jackson Labs (Stock #010905). We obtained female MF1 mice and males with the FCG mutation on the Y-chromosome and *Sry* insertion from Dr. Art Arnold (UCLA). Each FCG lines produced four groups: phenotypically female mice with the XX (FXX) or XY (FXY) chromosome, and phenotypically male mice with the XX (MXX) or XY (MXY) chromosome. The colonies were maintained in humidity and temperature-controlled conditions of 23°C on a 12:12 light/dark cycle (lights off at 1700). Food (phytoestrogen-free Purina #2020 chow) and water were provided *ad libitum*. At weaning (21–23 days of age), mice were housed in same-sex groups of two or three individuals and switched to a 12:12 reversed dark/light cycle (lights off at 12pm). Mice were between 60–90 days old at the time of their surgeries.

### AGD Measurement

AGD was measured on postnatal day 10 using an electronic caliper. Two independent experimenters carried out the measurements with high inter-rater agreement. Group sizes within the B6 line were *n* = 66 for females and *n* = 81 for males. Group sizes within the FCG B6 line were *n* = 72 for XX females, *n* = 91 for XY females, *n* = 91 for XX males, and *n* = 72 for XY males. AGD of mice that did not survive to weaning were omitted from the data.

### Gonadectomies, jugular catheterization, and catheter maintenance

Mice were gonadectomized to remove any group differences in circulating levels of gonadal hormones. All surgeries (gonadectomy, sham, and jugular catheterization) were performed under either isoflurane (B6) or ketamine/xylazine (100/10 mg/kg, MF1) anesthesia. MF1 mice required ketamine/xylazine due to lower sensitivity to isoflurane. Both strains received Anjeso (meloxicam, 5 mg/kg, Henry Schein, Melville, NY) for analgesia immediately after surgery. After gonadectomy or sham surgery, mice were placed in pairs with novel cage mates.

After a period of at least two weeks, all mice received jugular catheters using methods previously described (Le et al. 2023). Catheters consisted of polyurethane tubing (0.043 cm I.D. × 0.068 cm O.D.) attached to single-channel vascular access buttons (25GA) and secured with protective aluminum caps (Instech Labs, Plymouth Meeting, PA). Catheters were flushed with saline before and after each daily testing session. If abnormal resistance was encountered during flushing or self-administration behavior deviated from baseline, patency was assessed. Patency was confirmed by blood return or, if absent, by the loss of righting reflex within 3 seconds following infusion of methohexital sodium (10 mg/kg). Data collection ceased for any mouse that failed the patency test.

### Testing Apparatus

After catheter surgery, mice were individually housed in a modified home cage containing a metal vertical plate (see Martini et al., 2020). During testing, the plate was replaced with an identical one that contained 2 nose-poke holes and a housing light. This allowed the animals to be tested in their home cage and for further habituation to the testing apparatus.

### Drugs

Cocaine-HCl was obtained from the National Institute of Drug Abuse and dissolved in sterile 0.9% physiological saline. Infusion duration was adjusted daily based on individual body weight (2 s/27.5 g) to maintain a consistent mg/kg cocaine dose.

## General Self-Administration Procedures

### Acquisition

Acquisition testing began 4–6 days after catheter surgery. Mice were moved to the testing room at least one hour prior to testing and were tested for up to two hours each day during the dark phase of the reversed L:D cycle. Testing was conducted under a fixed-ratio 1 (FR1) schedule with sessions occurring daily for 12 consecutive days. Each session began with the illumination of a house light and cue light within the active (reward) hole and a priming infusion of cocaine. A nose-poke response in the active hole resulted in a cocaine infusion, followed by a 30-s time-out during which both lights were off. Active and inactive nose-poke responses were recorded throughout each session, but only active responses outside of the time-out had a programmed consequence (i.e., delivery of cocaine). Acquisition was defined as the first three consecutive sessions during which ≥ 70% of responses occurred in the active hole and intake averaged ≥ 10 mg/kg/day (Martini et al. 2014).

### Motivation

Motivation for cocaine was assessed in mice that met the acquisition criteria and had patent catheters. Progressive ratio testing began 2 days after the last acquisition session, ran daily for three sessions, and ended after either 4 hours or if no infusion had occurred within one hour. The cocaine dose used in PR matched the final dose used during acquisition as detailed in each experiment. The response requirement to earn an infusion escalated after each rewarded nose-hole poke according to the following series: 1, 2, 4, 6, 9, 12, 16, 20, 25, 30, 36, 42, 49, 56, 64, 72, 81, 90, 100, 110, etc. The breakpoint was defined as the final ratio completed and served as an index of motivation (Arnold and Roberts 1997).

## Experiment-Specific Self-Administration Procedures

### Experiment 1: B6 Mice

Adult WT B6 mice, between 60–90 days of age, were randomly assigned to gonadectomy or to sham. At the time of surgery, all sham surgery mice (*n* = 14 females, SF; *n* = 13 males, SM) and half of the gonadectomized mice received an implant filled with cholesterol (Ch) (*n* = 13 females, GF; *n* = 15 males, GM). The other gonadectomized mice received an implant filled with estradiol-17β (E2) (*n* = 14 females, EF; *n* = 16 males, EM). Implants were prepared using Silastic tubing (8–10 mm segments; 1.57 mm I.D. X 2.41 mm O.D.) sealed at one end with medical adhesive (Factor II, Lakeside AZ) and filled with either E2 diluted 1:1 with Ch or Ch alone, tamped to a final packed length of 5 mm. The open end was sealed and then trimmed to leave 1 mm of Silastic on each end. Based on earlier work in our lab (Le et al., 2023), we know that the E2 implants produce high (female) physiological levels of E2 in plasma. Implants were inserted subcutaneously on the dorsal surface of the back above the tail. At the end of the study, all implants were checked to make sure they were present and were not empty.

Acquisition testing occurred with a cocaine dose escalating across the 12-day period: 0.3 mg/kg for sessions 1–4, 0.6 mg/kg for sessions 5–8, and 1.0 mg/kg for sessions 9–12. This dose was selected based on previous results that gave modest acquisition (Martini et al. 2020). Sessions ended after two hours or when the maximum number of infusions were obtained (66 infusions for 0.3 mg/kg; 33 infusions for 0.6 mg/kg and 20 infusions at the highest dose), whichever occurred first. Mice that lost catheter patency before acquisition were excluded from all statistical analyses. Those that lost patency after acquisition were included in the percent acquisition and days to acquire data sets. The group size for each of the groups is as follows: SF *n* = 12, SM *n* = 11, GF *n* = 12, GM *n* = 12, EF *n* = 9, EM *n* = 13.

Motivation testing occurred daily for three sessions as detailed above using the 1.0 mg/kg dose of cocaine. Only mice with confirmed patency for at least 1 PR session were included in this analysis. Group sizes were, with *n*’s of mice that only finished 1 session in parentheses: SF *n* = 10(1), SM *n* = 9(2), GF *n* = 10(1), GM *n* = 10(3), EF *n* = 7(0), EM *n* = 12(3).

### Experiment 2: FCG B6 mice

Adult FCG B6 mice were between 60–90 days of age at the start of the study. All mice were gonadectomized and did not receive hormone replacement. Group sizes before acquisition were as follows: FXX *n* = 13, MXX *n* = 15, FXY *n* = 13, MXY *n* = 12. Acquisition testing occurred as detailed in experiment 1, but used a lower dose escalation procedure. We did this because our previous findings with B6 FCG mice under a 0.3/0.6/1.0 mg/kg escalation schedule led to nearly all mice acquiring just before or just after the highest cocaine dose (Le et al., 2023; Martini et al., 2020). Mice instead received 0.2 mg/kg cocaine for sessions 1–4, 0.4 mg/kg for sessions 5–8, and 0.6 mg/kg for sessions 9–12. The sessions ended after two hours or when the maximum number of infusions were obtained (44 infusions for 0.2 mg/kg; 39 infusions for 0.4 mg/kg and 33 infusions at the highest dose). The group size for each of the groups was as follows: FXX *n* = 12, MXX *n* = 14, FXY *n* = 12, MXY *n* = 7.

Motivation testing and criteria remained the same as Experiment 1 but used the 0.6 mg/kg dose of cocaine. The final group sizes were as follows, with n’s of mice that only finished 1 session in parentheses: FXX *n* = 12(3), MXX *n* = 13(5), FXY *n* = 12(3), MXY *n* = 7(0).

### Experiment 3: FCG MF1 mice

The same procedure was used as Experiment 2 with the MF1 strain of FCG mice. Group sizes before acquisition were as follows: FXX *n* = 14, MXX *n* = 13, FXY *n* = 19, MXY *n* = 13. Group sizes after acquisition were as follows: FXX *n* = 11, MXX *n* = 9, FXY *n* = 15, MXY *n* = 10. For PR testing final group sizes were as follows: FXX *n* = 10(3), MXX *n* = 8(1), FXY *n* = 13(6), MXY *n* = 10(5).

## Molecular Procedures

### Tissue collection

In all experiments, 24 hours after the final PR test, mice were given a 10 mg/kg dose of cocaine (intra-peritoneal injection) 20–30 minutes before euthanasia under either isoflurane (B6) or ketamine/xylazine anesthesia. Brains were removed and coronal sections were made using a 0.5 mm coronal brain matrix (Braintree Scientific, Pembroke, MA). NAc (shell and core) was collected using a 1.5 mm (ID) biopsy punch (World Precision Instruments, Sarasota, FL). The coordinates for the region were Bregma 0.20–1.20 mm. These were based on the adult mouse atlas (Paxinos and Franklin 2001). Tissues were placed in RNA-free microfuge tubes, frozen rapidly on dry ice, and stored at −80°C.

### Quantitative PCR

mRNA expression of *Tlr7* and *Tlr8*, two of the genes duplicated on the Y chromosome, were determined in the NAc. For each group, 5–6 mice were randomly selected from those that completed self-administration testing. RNA isolation was conducted using an RNeasy Lipid Tissue Kit (Qiagen, Germantown, MD) following the manufacturer’s protocol. cDNA templates were prepared using an AffinityScript qPCR cDNA Synthesis Kit (Agilent Technologies, Santa Clara, CA) according to the manufacturer’s protocol. The ABI StepOnePlus real-time PCR system was used to perform qRT-PCR using SYBR^®^Green-Based Detection (Applied Biosystems, Carlsbad, CA). In these assays, all samples were normalized to the mouse housekeeping gene *Gapdh.* Oligonucleotide primers (Supplemental Table 1) were designed using consensus sequences and Blast from the NCBI genomic alignment database and were synthesized by Invitrogen (Carlsbad, CA).

### Y-Chromosome Sequencing

WGS was generated for cocaine-naïve, experimentally naïve mice to examine potential structural and sequence differences on the Y chromosome. Tail tip samples were collected and pooled from the following groups (n = 5 per group): B6 FCG females XX and XY, B6 FCG males XX and XY and, B6 females and males. DNA was then extracted and 150 bp paired-end WGS was conducted on the 6 samples on an Illumina NovaSeq yielding 351–541 million read pairs. Three of these samples were used to generate genome assemblies: FXY, MXY and B6 M.

The reads were quality and adapter trimmed using fastp (v 0.21.0) (Chen et al. 2018). The GATB Minia assembly pipeline was used with the --no-scaffolding and --no-error-correction flags to assemble the three samples (Chikhi and Rizk 2013; Drezen et al. 2014). As can be seen in Supplemental Table 2, the assemblies are relatively complete in terms of total length (Gb) when compared with that of GRCm39 primary genome size (from Ensembl release 110 : Mus_musculus.GRCm39.dna.primary_assembly.fa) which is 2.73 Gb. However, the largest contig lengths and the N50 values are small, which is not unexpected in generating assemblies from short-reads.

The assembly contigs were aligned to the GRCm39 primary genome assembly using minimap2 with flags -ax asm5 (version 2.24-r1122) and the resulting files were converted to sorted bam files using samtools (version 1.12) (Li 2018; Danecek et al. 2021). The contig alignments for genes of interest on the Y chromosome (*Ddx3y, Eif2s3y, Kdm5d, Uba1y, Usp9y, Uty, Zfy1* and *Zfy2*) and the X pseudoautosomal region (*Asmt, Mid1*, and *Sts*) were examined using the Integrative Genomics Viewer (IGV) (Robinson et al. 2011). Only the candidate gene regions that had an aligned contig in all three assemblies were included in the comparisons.

To examine the read depth for two regions of the genome for FXY, MXY and B6 M, the trimmed reads were mapped to GRCm39 using bwa mem (version 0.7.17) (Li 2013). The first region of interest was for a single nucleotide polymorphism (SNP) observed in *Zfy2* for FXY and MXY. The second region was for a reported translocation in XY FCG samples (Panten et al. 2024). The read coverage for this region can be seen in Supplemental Fig. 1.

The Illumina reads were also used for variant calling, which was performed using GATK (McKenna et al. 2010). The *Zfy2* SNP was in the FXY and MXY VCF files, but not in the B6 M VCF file. The Ensembl Variant Effect Predictor (VEP, version 112) was used with GRCm39 to annotate the FXY VCF file and evaluate the predicted impact of the *Zfy2* SNP (McLaren et al. 2016). The package ‘karyoploteR’ was used to plot the read depths in R (Gel and Serra 2017; R Core Team 2025).

### Statistical Analyses

Survival analyses for acquisition over the 12-day testing period were conducted using the Cox proportional hazards regression model to examine the association between sex (gonadal male or female) and either hormone status (Sham-Ch, GDX-Ch, GDX-E2; Experiment 1) or SCC (XX or XY, and gonadal sex; Experiments 2 and 3). Combined analysis between Experiments 2 and 3 included strain (B6 or MF1) as a factor. No interactions between each predictor and the log of survival time were found for all analyses, indicating the proportional hazards assumption was met. All other self-administration analyses were limited to the mice that acquired cocaine self-administration and had patent catheters.

Two-factor ANOVA was used to compare number of days to acquire between gonadal sex and either hormone group (Experiment 1) or SCC (Experiment 2 and 3). Two-way repeated measures ANOVA was used to compare sex and hormone group (Experiment 1) or SCC and gonadal sex (Experiments 2 and 3) for differences in intake (mg/kg/day), active nose-poke responses, proportion of active over total responses, total responses, and inactive responses over the 12 days of acquisition testing. Three-way ANOVA was used for combined analyses of Experiments 2 and 3 after collapsing across day given that there were no significant overall or interactive effects of day. To hone in on our predicted differences between the two strains, follow-up two-way ANOVA was conducted within each of the SCC groups (XX and XY). Day effects were explored during each of the three dose-phases of acquisition (low, days 1–4; medium, days 5–8; high, days 9–12) either as average (to explore overall effects of day) or over the four days within each phase (to explore interactive effects of day and SCC/sex). All post-hoc comparisons were Bonferroni-corrected (Benjamini and Hochberg 1995).

Mixed effects models were used to analyze PR data since not all mice remained patent across the 3-day testing period. Separate analyses were used for infusions earned, active nose pokes, and inactive nose pokes using day, sex and hormone (Experiment 1) or SCC (Experiments 2 and 3) as between-subject fixed factors using the Variance Components procedure. Day was also a repeated measure with diagonal covariance structure. Holm-Bonferroni corrections were conducted for post hoc comparisons. AGD was compared between genotype (normal B6, XX B6 and XY B6) using one-way ANOVA for each sex.

Gene expression analysis was performed using Bonferroni-corrected two-sample t-tests for Experiment 1 when Levene’s test for equality of variances was met. Welch’s ANOVA was used otherwise. Two-way ANOVAs were used for Experiments 2 and 3. Correlation analyses between gene expression and behavioral measures were conducted using Spearman’s rho. Grubb’s test was used to find and remove a MF1 XY female outlier.

A priori power analysis indicated that a total sample size of 16 would be sufficient to detect a sex × SCC interaction in a rmANOVA with 12 time points (ICC = 0.015), assuming α = 0.05 and desired power = 0.80, yielding an actual power of 0.91. Effect size estimates were derived from our previous study (Le et al. 2023), which reported partial η^2^ = 0.16 (Cohen’s f = 0.44). Sample size calculations were conducted using G*Power 3.1 (Faul et al. 2007). All other analyses were conducted using Statistical Package for Social Sciences (SPSS) for Windows ver.29 (Armonk, NY) and/or Number Cruncher Statistical Systems (NCSS) 24 for Windows (Kaysville, UT). Alpha was set to 0.05.

## Results

### Experiment 1. No Effects of Sex or Hormonal Status on Vulnerability to Cocaine in Wild-Type B6 Mice

**Figure 1:**
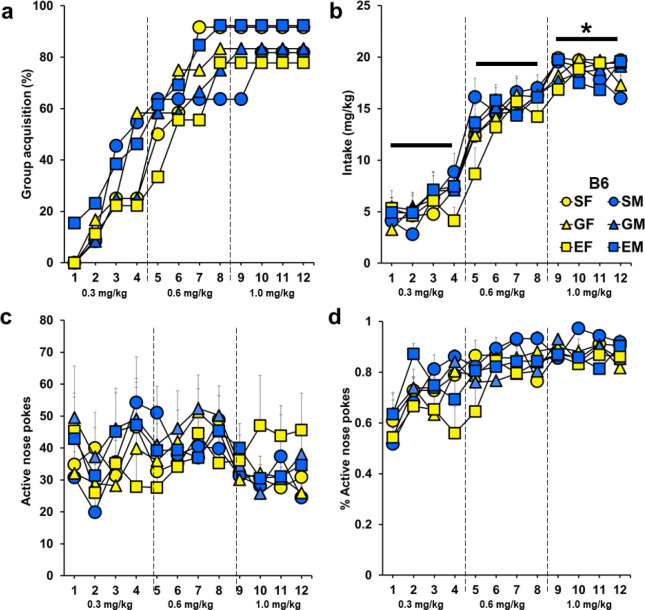
Experiment 1: Cocaine self-administration in B6 mice. Mice were tested using an FR1 schedule with escalating doses of cocaine (0.3 mg/kg/infusion for days 1–4, 0.6 mg/kg/infusion for days 5–8, 1.0 mg/kg/infusion for days 9–12). a) Percent of each group reaching cocaine self-administration acquisition criteria. b) Amount of cocaine taken daily (Mean + SEM). *Cocaine intake was greatest in all groups at the end of the study versus the beginning and middle (days 1–4 and 5–8 versus 9–12), *p* < 0.001. c) Active nose-poke responses (Mean + SEM). d) Ratio of active nose pokes over the total nose pokes (Mean + SEM) for each group over the FR1 self-administration test. Yellow symbols represent females and blue symbols represent male mice. Circle symbols represent sham animals, triangles are gonadectomized animals, and squares are animals given E2 implant replacement. SF = sham female with control implant (*n* = 10), GF = gonadectomized female with control implant (*n* = 9), EF = gonadectomized female with E2 implant (*n* = 7), SM = sham male with control implant (*n*= 9), GM = gonadectomized male with control implant (*n* = 10), EM = gonadectomized male with E2 implant (*n* = 12)

Maximal and rapid rates of acquisition were observed In B6 mice, with over 75% of the mice within each of the groups acquiring cocaine self-administration within the 12-day testing period (Fig. 1a). There were no significant overall or interactive effects of sex (*p* = 0.74) or hormone status (*p’s* = 0.74, 0.99, 0.99). Number of days to acquire also did not differ between males and females or by hormonal status, nor was there an interaction (*p’s* = 0.76, 0.95, 0.40).

Daily cocaine intake (mg/kg) did not differ by sex or hormonal status nor was there an interaction (*p’s* = 0.53, 0.82, 0.86). There was a significant effect of day (*F*(11, 572) = 125.49, *p* < 0.001), which reflects greater cocaine intake at the end of acquisition testing (days 9–12), when the dose of cocaine was highest, as compared to both the beginning (days 1–4; *p* < 0.001) and middle (days 5–8, *p* < 0.001) phases (Fig. 1b). No day by sex or day by hormone group effects were found (*p*’s = 0.07, 0.73). There were also no overall or interactive effects of sex or hormonal status for active responses or percent active/total responses (Fig. 1c–d). Similar results were observed for total responses and inactive responses (Supplemental Fig. 2a-b).

The number of infusions obtained under the PR schedule did not differ by sex or hormone status, nor was there an interaction (*p’s* = 0.99, 0.21, 0.74, Supplemental Fig. 3a). There was also no overall day effect (*p* = 0.24) or interactive effects of day by sex (*p* = 0.14) or hormone treatment (*p* = 0.76). Similar results were observed for active and inactive responses (Supplemental Fig. 3b-c).

### Experiment 2. SCC Affects Vulnerability to Cocaine in FCG B6 Mice

**Figure 2:**
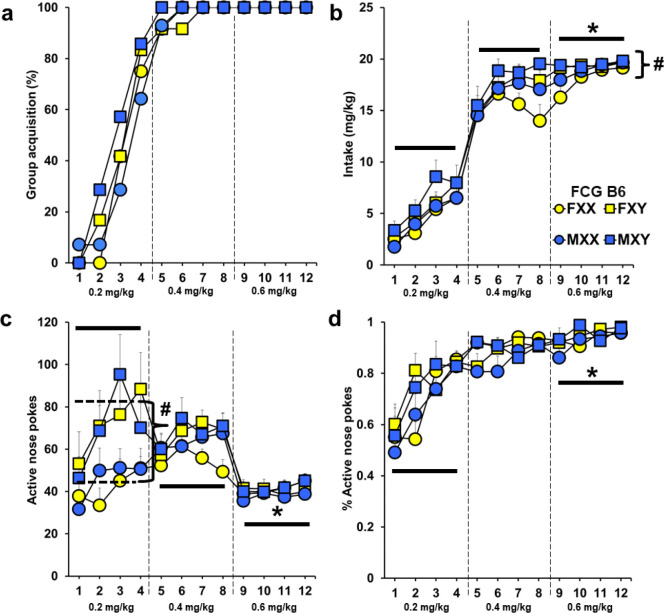
Experiment 2: Cocaine self-administration in B6 FCG gonadectomized mice. Mice were tested using FR1 schedule with escalating doses of cocaine (0.2 mg/kg/infusion for days 1–4, 0.4 mg/kg/infusion for days 5–8, 0.6 mg/kg/infusion for days 9–12). a) Percent of each group reaching cocaine self-administration acquisition criteria. b) Amount of cocaine taken daily (Mean + SEM). *Cocaine intake was greatest in all groups at the end of the study versus the beginning and middle (days 1–4 and 5–8 versus 9–12), *p* < 0.001. #XY mice took significantly more cocaine than XX mice, *p* < 0.05. c) Active nose-poke responses (Mean + SEM). *Responding was significantly lowest in all groups at the end of the study versus the beginning and middle (days 1–4 and 5–8 versus 9–12), *p* < 0.001. #XY mice had significantly higher intake than XX mice between days 1–4, *p*< 0.01. d) Ratio of active nose pokes over the total nose pokes (Mean + SEM). *Significantly higher at end of study versus the beginning (days 1–4 versus 9–12), *p* < 0.001. Yellow symbols represent females and blue symbols represent males. All mice were gonadectomized. Circles represent XX genotype mice, while squares represent XY mice. B6 FXX are gonadally female mice with an XX chromosome (*n* = 12), B6 FXY are gonadally female mice with an XY chromosome (*n* = 12), B6 MXX are gonadally male mice with an XX chromosome (*n* = 13), B6 MXY are gonadally male mice with an XY chromosome (*n* = 7)

Despite the lower cocaine doses in this experiment as compared to Experiment 1, all of the B6 FCG mice in this experiment acquired cocaine self-administration during the 12-day testing period. All four groups also acquired rapidly and there were no significant effects of sex, SCC, or an interaction (*p*’s = 0.94, 0.78, 0.47). In addition, there were no differences by sex or SCC, nor were there any significant interactions on time to acquisition (*F*(1, 40) = 0.26, 1.37, 0.53 respectively; Fig. 2a).

There was a significant effect of SCC on cocaine intake caused by XY mice taking more cocaine than XX mice (*F*(1, 440) = 3.95, *p* < 0.05; Fig. 2b). Intake was not affected by gonadal sex (*F*(1, 440) = 1.35) nor was an interaction found (*F*(1, 440) = 0.001). A robust effect of day was present (*F*(11, 440) = 238.03, *p* < 0.001), which reflects higher intake at end of acquisition testing (days 9–12) as compared to both days 1–4 and 5–8 (*p* < 0.001). Neither sex or SCC interacted with day (*p*’s = 0.74, 0.41).

There was also a main effect of SCC on nose pokes into the active hole (*F*(1, 440) = 7.68, *p* < 0.01, Fig. 2c). As with intake, more responses were displayed by XY than XX mice. This was particularly apparent at the beginning of acquisition testing (interaction of SCC by day, *F*(11, 440) = 2.92, *p* < 0.05), with XY mice having higher active pokes than XX mice during sessions 1–4 (*F*(1, 120) = 7.30, *p* < 0.05), but not during sessions 5–8 or 9–12 (*p* > 0.05). There was also a significant effect of day (1,440) = 10.61, *p* < 0.001) which reflects lower responses at the end of acquisition testing as compared to the beginning and middle phases (days 1–4 and 5–8 versus 9–12; *F*(1, 40) = 27.81, *p* < 0.01). No effect of gonadal sex was present nor were there any interactions (*p*’s = 0.71, 0.74). Similar overall and interactive effects of SCC and day were observed for total nose pokes, although the SCC effect within days 1–4 was less robust and only evident as a trend (day by SCC interaction; *F*(11, 440) = 1.99, *p* < 0.05 on days 3 and 4; Supplemental Fig. 4a).

Percentage of active/total nose pokes did not reveal any significant effects of sex, genotype or their interaction (*p*’s = 0.46, 0.15, 0.39, Fig. 2d). There was a significant effect of day (*F*(11, 440) = 26.34, *p* < 0.001) with higher percentages observed at the end of testing as compared to the beginning (days 1–4 versus 9–12; *F*(1, 40) = 94.97, *p* < 0.001). There were also no overall or interactive effects of SCC or gonadal sex for inactive nose pokes, however, a significant effect of day was found (*F*(1, 440) = 13.97, *p* < 0.001). Mice poked less on the end of testing versus the beginning and middle phases (*F*(1, 33) = 33.10, *p* < 0.001; Supplemental Fig. 4b).

During PR, an overall effect of SCC was found (*F*(1, 88.78) = 5.57, *p* < 0.05) for the number of infusions earned over the 3-day testing period. XY mice received more rewards than XX mice (Supplemental Fig. 5a). No effects of sex or day were found, nor were there any interactions (*p*’s = 0.71, 0.12, 0.73). While there were no overall or interactive effects of these factors in the analysis of active nose-poke numbers, there was a significant effect of SCC on inactive nose pokes (*F*(1, 79.90) = 5.70, *p* < 0.05; Supplemental Fig. 5b-c). Interestingly, XX mice had greater incorrect responses than XY mice. An effect of day was also noted (*F*(2, 54.47) = 4.71, *p* < 0.05). Post-hoc analysis revealed that PR-day 1 had more responding than day 3 (*p* < 0.01). No effects of sex or interactions were noted (*p*’s = 0.36, 0.12).

### Experiment 3. Modest Effect of Gonadal Sex on Vulnerability to Cocaine in MF1 Mice

**Figure 3:**
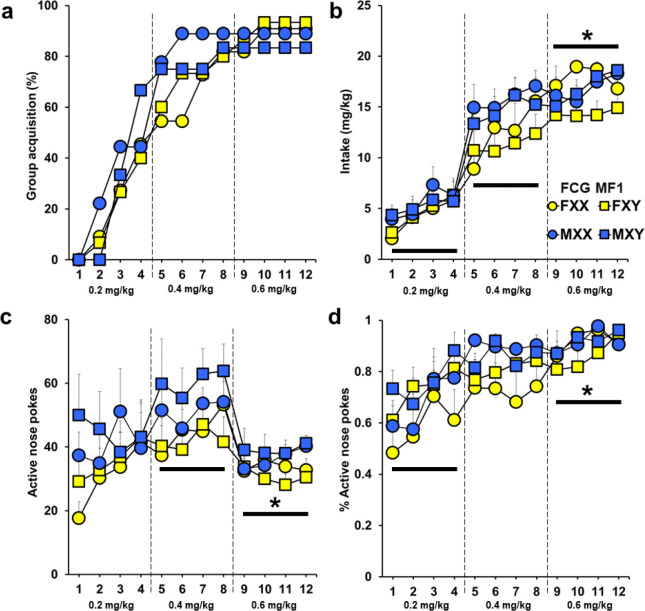
Experiment 3: Cocaine self-administration in gonadectomized FCG MF1 mice. Mice were trained using an FR1 with escalating doses of cocaine (0.2 mg/kg/infusion for days 1–4, 0.4 mg/kg/infusion for days 5–8, 0.6 mg/kg/infusion for days 9–12). a) Percent of each group reaching cocaine self-administration acquisition criteria. b) Amount of cocaine taken daily (Mean + SEM). *Cocaine intake was greatest in all groups at the end of the study versus the beginning and middle (days 1–4 and 5–8 versus 9–12), *p* < 0.001. c) Active nose-poke responses (Mean + SEM). *Significantly less responding during the end of study versus the middle (days 5–8 versus 9–12), *p* < 0.01. d) Ratio of active nose pokes over the total nose pokes (Mean + SEM) for each group over the FR1 self-administration test. *Proportion of correct responses was significantly higher at end of the study versus the beginning (days 1–4 versus 9–12), *p* < 0.001. All mice were gonadectomized. MF1 FXX (*n* = 11), MF1 FXY (*n* = 14), MF1 MXX (*n* = 9), MF1 MXY (*n* = 12)

At least 80% of each MF1 FCG group acquired self-administration cocaine by the end of the testing period. There were no effects of sex, SCC, or an interaction found in acquisition rate (*p*’s = 0.58, 0.90, 0.70). In addition, we did not find group differences by sex or SCC, nor were there any interactions in time until acquisition (*F*(1, 37) = 3.04, 0.00, 0.16, respectively; Fig. 3a).

Cocaine intake also did not differ by sex or SCC, nor were there interactions (*p*’s = 0.12, 0.38, 0.62; Fig. 3b). There was a significant effect of day (*F*(11, 407) = 66.19, *p* < 0.001), which reflects greater intake at the end versus beginning and middle of acquisition testing (days 1–4 and 5–8 versus 9–12; *p* < 0.001), but no interaction between sex or SCC with day (*p’s* = 0.92, 0.09). Active nose-poke responses tended to be greater for males than females (*p* = 0.08; Fig. 3c). Sex chromosome complement was not influential (*p* = 0.65) and had no interaction with gonadal sex (*p* = 0.57). We noted an effect of day (*F*(11, 407) = 4.47, *p* < 0.001), however, responses at the end of the sessions were only lower compared to the middle sessions’ (days 5–8 versus 9–12; *F*(2, 74) = 7.81, *p* < 0.001). No interactions between day and sex or day and SCC were found (*F*(11, 407) = 0.74, 0.48, respectively).

The proportion of active responses relative to total responses was unaffected by sex or SCC, and no interactions were observed (*p’s* = 0.14, 0.40, 0.75; Fig. 3d). A main effect of day was present (*F*(11, 407) = 11.18, *p* < 0.001). This was reflective of a higher proportion of correct responses during days 9–12 versus 1–4 (*F*(2, 74) = 179.818, *p* < 0.001). There were no interactions of day between either factor (*p’s* = 0.67, 0.13 for sex and SCC). Males tended to poke more overall than females (*F*(1, 407) = 3.10, *p* = 0.09; Supplemental Fig. 6a). Sex chromosome complement did not have an effect on total nose pokes, nor were there any interactions (*F*(1, 407) = 0.26, 0.50, respectively). There was a main effect of day (*F*(11, 407) = 4.94, *p* < 0.001) in which total responding was lowest during the high dose period versus the middle dose period (days 5–8 versus 9–12; *F*(2, 74) = 8.28, *p* < 0.001). Interactions between day and sex or day and SCC were not significant (*p’s* = 0.30, 0.93). No effects or interactions were noted for inactive nose-poke responses (Supplemental Fig. 6b).

Number of infusions earned during PR did not reveal an effect of sex or SCC, nor was there an interaction (*p’s* = 0.13, 0.78, 1.78; Supplemental Fig. 7a). Similar results for active nose pokes were found, however, a trend for an interaction between sex and SCC was noted (*F*(1, 56.17) = 3.31, *p* = 0.07; Supplemental Fig. 7b). An effect of sex was found for inactive nose pokes (*F*(1, 84.41) = 4.9, *p* < 0.05) caused by males incorrectly poking more than females (Supplemental Fig. 7c). An effect of day was also noted (*F*(2, 44.25) = 4.10, *p* < 0.05) caused by day 1 being different from day 3 (*p* < 0.01). No effect of SCC or interactions were present (*p’s* = 0.39, 0.36).

### Strain Differences in Acquisition and Motivation for Cocaine (B6 > MF1)

**Figure 4:**
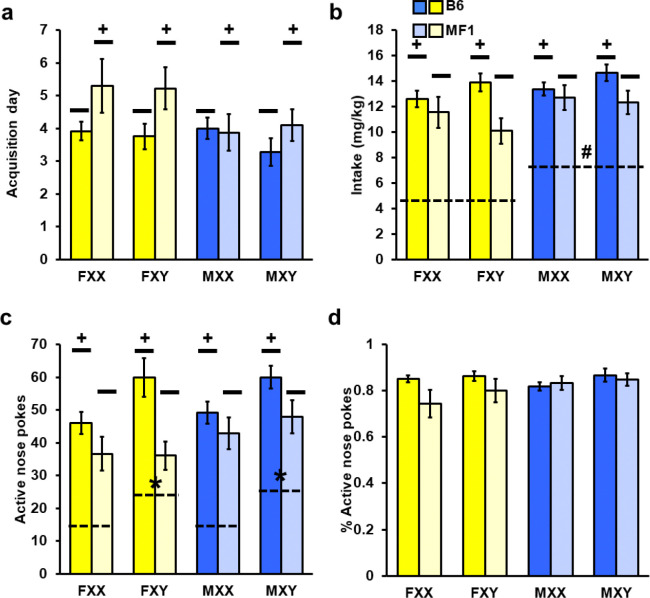
Summary of Experiments 2 and 3. a) Daily average (Mean +/− SEM) acquisition day. +B6 mice acquired sooner than MF1 mice, *p* < 0.05. b) Daily average cocaine intake by day. +B6 mice took significantly more cocaine than did MF1 mice, *p* < 0.01. #Males received more cocaine than did females, *p* < 0.05. c) Daily average (Mean +/− SEM) active nose pokes. +B6 mice responded in the active nose poke hole more often than MF1 mice, *p* < 0.001. *XY mice displayed more active hole pokes than did XX mice, *p* < 0.05. d) Daily average (Mean +/− SEM) ratio of active nose pokes over total nose pokes. Yellow bars represent females and blue bars represent males. Darker bars are B6 mice while lighter bars are MF1 mice

We conducted Experiments 2 and 3 simultaneously in order to compare behavior between the two strains. While there were no significant effects of sex or SCC on acquisition rate (*p* = 0.08, 0.53, respectively), there was a significant effect of strain (*Wald* χ^2^(1) = 7.86, *p* < 0.01; Figs. 2a and 3a) with B6 mice acquiring faster than MF1 mice. Planned comparison within XX and XY mice revealed a significant effect within XY mice (*Wald* χ^2^(1) = 5.27, *p* < 0.05) but not XX mice (*p* = 0.20), indicating that this strain effect was driven by XY mice regardless of gonadal sex. Mean days to acquire self-administration also differed by strain (*F*(1, 77) = 4.85, *p* < 0.05), although follow-up comparison within SCC only displayed a trend in XY mice (*F*(1, 42) = 3.51, *p* = 0.07). (Fig. 4a). There was also a trend for a sex effect in the overall analysis of days to acquire (*F*(1, 77) = 3.22, *p* = 0.08), with males tending to acquire in fewer days than females.

For average cocaine intake we noted an overall strain effect (*F*(1, 77) = 10.00, *p* < 0.01) caused by B6 mice taking more cocaine than MF1 mice (Fig. 4b). Planned comparison within XX and XY mice revealed this effect was again driven by XY mice (*F*(1, 38) = 11.47, *p* < 0.01) and not XX mice (*p* = 0.34). We also found an overall sex effect (*F*(1, 77) = 3.96, *p* < 0.05) caused by males taking more cocaine than females, but this was lost when split by SCC. No effect of SCC was found in the overall analysis of intake (*p* = 0.78). Active nose pokes had similar results, showing an effect of strain (*F*(1, 77) = 14.97, *p* < 0.001; Fig. 4c) again caused by B6 poking more than MF1 mice. Planned comparison between XX and XY revealed the effect was again only found in XY mice (*F*(1, 38) = 11.73, *p* < 0.05) but not in XX mice (*p* = 0.10). An effect of SCC was also found (*F*(1, 77) = 4.86, *p* < 0.05) with XY groups poking more than XX groups. No interactions for strain by sex, strain by SCC, or sex by SCC were noted (*p*’s = 0.27, 0.13, 0.84). No overall effects or interactions were found for the proportion of active pokes over total responses (p > 0.05, Fig. 4d) or inactive responses (*p* > 0.05, Supplemental Fig. 8a).

A significant strain effect was found in total responses (*F*(1, 77) = 13.86, *p* < 0.001) due to B6 mice responding more overall than MF1 mice. Planned comparisons by SCC showed an effect of strain in both XY (*F*(1, 38) = 9.34, *p* < 0.001) and XX mice (*F*(1, 39) = 4.47, *p* < 0.05). Both sex and SCC showed trends (*F*(1, 77) = 3.30, 3.31, *p* = 0.07 for both, respectively). No interactions between strain, sex, or SCC were found (*p* = 0.49, 0.28, 0.81, Supplemental Fig. 8b).

Analysis of rewards earned during PR also revealed only a strain effect (*F*(1,149.67) = 26.56, *p* < 0.001; Supplemental Fig. 8c-d). Active nose-poke responses showed similar results, with B6 mice responding more than MF1 mice (*F*(1,156.55) = 17.99, *p* < 0.001; Supplemental Fig. 8d).

### AGD is Reduced in B6 FCG mice

**Figure 5:**
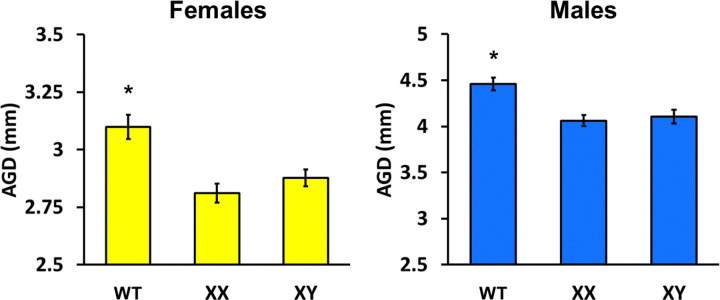
Anogenital distance on post-natal day 10 in normal B6 versus B6 FCG mice. *In both sexes, normal B6 mice had larger anogenital distance than B6 backcrossed into FCG, *p* < 0.001. WT are wild-type B6 mice. XX are mice with an XX chromosome. XY are mice with an XY chromosome.

This is the first comparison, to our knowledge, of AGD in wild-type B6 mice versus B6 FCG mice. In females, we found a significant overall effect of genotype (*F*(2, 228) = 11.15, *p* < 0.001) with WT B6 mice having greater AGD than both XX and XY variants (*p* < 0.001 for both, Fig. 5). The same effect was found in males (*F*(2, 239) = 11.44, *p* < 0.001) where again C57 AGD was greater than both FCG genotypes (*p* < 0.001 for both). Within each sex, no differences between XX and XY FCG mice were present (*p* = 0.26 in females, *p* = 0.65 in males).

### Sequencing Reveals Single Nucleotide Polymorphism (SNP)

**Figure 6:**
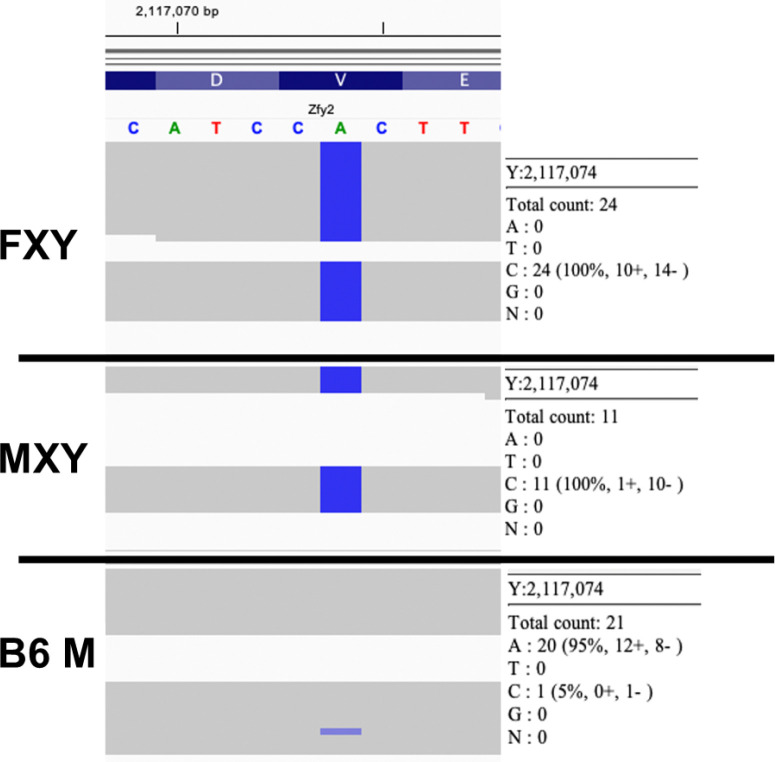
The figure shows the number of reads that have either an ‘A’ or a ‘C’ for *Zfy2* at 2,117,074 bp. The figure was generated using IGV and the reads for each sample are displayed in ‘Squished’ format. The reads in FXY (count: 24) and in MXY (count: 11) are all ‘C’ at 2,117,074 bp. B6 M (count: 21) had 1 read with a ‘C’ and 20 reads with an ‘A’. The GRCm39 reference is ‘A’ at this position

In zinc finger protein Y-linked 2, *Zfy2*, found in exon 5 on the Y chromosome, we observed that the nucleotide at 2,117,074 bp was a ‘C’ for the FXY and MXY assembly contigs, whereas it was an ‘A’ in the B6 M contig as well as in GRCm39. Figure 6 showed that the majority of the reads in B6 M matched the nucleotide observed in GRCm39 (‘A’), whereas none of the reads in FXY or MXY matched GRCm39. This variant was also observed in the FXY and MXY VCF files, but not in the B6 M VCF File. VEP annotations were generated for the FXY sample and the consequence for the ‘A’ -> ‘C’ change is a nonsynonymous missense mutation V251G. The VEP impact of this SNP is ‘Moderate’, which is defined as a non-disruptive variant that might change protein effectiveness. The VEP annotation also included a SIFT predictive score as to whether the amino acid substitution V251G impacts the protein function (Ng 2003). The SIFT score labeled this SNP as deleterious.

To get a better sense of the local environment of this SNP, we examined the amino acid V251 for *Zfy2* using NetSurfP-3.0 and the RCSB PDB to examine the secondary structure and disorder. NetSurfP-3.0 shows that the SNP is in a coil, where the probability of the residue being disordered is 71% (Høie et al. 2022). When the structure of *Zfy2* was examined using the RCSB PDB (https://www.rcsb.org/sequence/AF_AFP20662F1#A) at amino acid position 251, it could be seen that the AlphaFold per-residue model confidence score was ‘Very low’ for this region (Berman et al. 2000; Jumper et al. 2021).

Given these annotations, one might say that based on amino acid physical properties, such as that which is used in SIFT, the SNP is deleterious. However, given that this SNP exists in a region of the protein which is predicted to be disordered, we caution that it is hard to assess the impact of this missense variant. Examination of all known mammalian sequences for *Zfy2* did not find any examples of this SNP (Blast search). This suggests the SNP in nonviable.

### mRNA for both Toll-like Receptors are Sensitive to SCC

**Figure 7:**
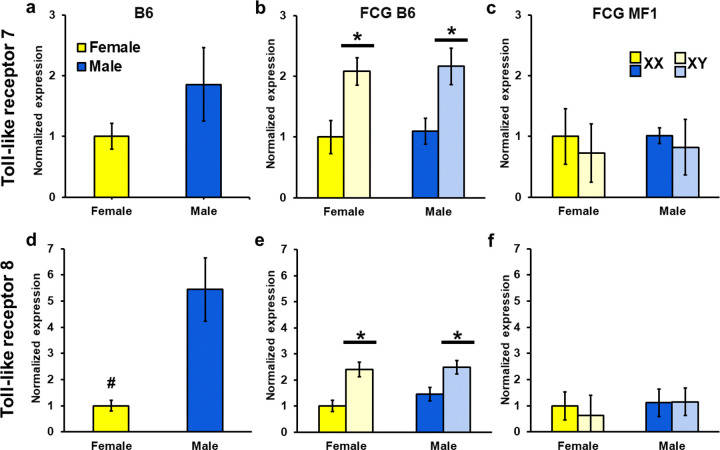
Relative mRNA expression levels for toll-like receptor 7 (*Tlr7)* and toll-like receptor 8 *(Tlr8)* in nucleus accumbens. For all comparisons the reference group was the XX females. Panels a and d are data from B6 mice, n=6 per group. Panels b and e are data from FCG B6 mice, n=6 per group. Panels c and f are data from FCG MF1 mice, n=5–6 per group. a) No differences between gonadectomized B6 males and females were noted. b) A sex chromosome complement effect was found in B6 FCG mice. *Mice with XY sex chromosome had more mRNA than XX, *p* < 0.05). c) No differences between gonadectomized MF1 FCG mice were noted. d) No significant differences between gonadectomized B6 males and females were noted. #A trend (*p* = 0.07) for a sex difference. e) *XY mice had higher mRNA expression than XX mice, *p*< 0.05. F) No differences were found in gonadectomized MF1 FCG mice. Darker bars are XX mice while lighter bars are XY mice

We compared expression of *Tlr7* between normal B6 males and females in Experiment 1 and found no effect of sex in gonadectomized mice (*t* = 1.39, Fig. 7a). In FCG B6 mice from Experiment 2, we found an effect of SCC (*F*(1, 20) = 16.00, *p* < 0.001). This was produced by XY mice which exhibited higher expression than XX mice (*p* < 0.05 Fig. 7b). We did not find any effects of sex (*F*(1, 20) = 0.14). No interactions between SCC and sex were found (*F*(1, 20) = 0.02). MF1 mice from Experiment 3 did not show any effects of SCC or sex on *Tlr7* mRNA, nor were there interactions (*F*(1, 19) = 0.06, 0.90, 0.04 respectively, Fig. 7c). *Tlr8* expression in gonadectomized B6 mice showed a trend for an effect of sex (*t* = 2.00, *p* = 0.07) with males tending to express more *Tlr8* than females (Fig. 7d). In FCG mice, B6 XY mice had more expression of *Tlr8* as compared with XX mice (*F*(1, 20) = 16.05, *p* < 0.05; Fig. 7e). No effect of sex or interactions were observed (*F*(1, 20) = 0.1.34, 0.93, respectively). Expression of *Tlr8* did not differ by sex or SCC in the MF1 mice, nor was there an interaction (*F*(1, 19) = 0.82, 0.31, 0.39, Fig. 7f).

To examine strain effects, data from Experiment 2 and 3 were analyzed together. No main effects of strain, sex, or SCC were found (*F*(1, 39) = 0.64, 0.17, 1.82, respectively). We noted a strain by SCC interaction (*F*(1, 39) = 8.79, *p* < 0.01). This interaction reflects the higher expression of *Tlr7* within B6 XY mice as discussed above, this was not found in the MF1 XY mice (*p* < 0.02). No strain by sex or sex by genotype interactions were found (*F*(1, 39) = 0.00, 0.01, respectively).

For expression of *Tlr8*, a strain by SCC interaction was found (*F*(1, 39) = 4.6, *p* < 0.05). Post-hoc analysis showed that this interaction was caused by the MF1 XX mice which had higher *Tlr8* expression than XX B6 mice (*p* < 0.05). No strain, sex, or SCC effects were noted, nor were there interactions of strain by sex or sex by SCC (*F*(1, 39) = 2.10, 1.72, 1.23, 0.14, 0.04).

### Correlations between Gene Expression and Behavior in FCG mice

**Figure 8:**
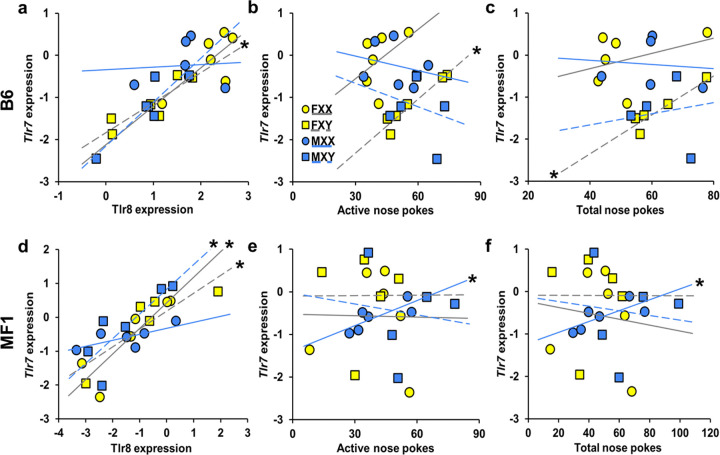
Correlations between mRNA expression in the nucleus accumbens and behavior in Experiments 2 and 3. Panels a, b, and c represent data from B6 FCG mice. Panels d,e, and f show data from MF1 FCG mice. a) *Significant correlation between mRNA levels of *Tlr7*and *Tlr8* in FXY B6 FCG mice*, p* < 0.05. b). *Significant correlation between mRNA levels of *Tlr7*and active nose pokes in FXY B6 FCG mice*, p* < 0.01. c) *Significant correlation between *Tlr7*mRNA and total nose pokes in FXY, *p* < 0.05. d) *Significant correlation between *Tlr7* and *Tlr8 expression*, in all groups of MF1 FCG mice except the MXX, *p* < 0.05. e) Correlations between *Tlr7 mRNA* and active nose-hole pokes are significant in MF1 MXX group, *p* < 0.01. f) *Significant correlation between *Tlr7* mRNA and total nose pokes, only in MXX, *p* < 0.05. Blue lines are trend lines for males while grey lines are trend lines for females. Solid lines represent XX groups while dashed lines represent XY groups

Since the 9 X-chromosome genes are only translocated to the Y-chromosome in B6 FCG mice (Panten et al. 2024), we examined B6 and MF1 mice separately. For both strains we calculated correlations between the individual levels of mRNA and mean behavioral measures. When we examined all 4 B6 SCC groups separately, only XY females had a strong positive correlation between *Tlr7* and *Tlr8* (*r* = 0.83, *p* < 0.05; Fig. 8a). While XY males showed a trend for this direction (*r* = 0.77, *p* = 0.07), neither male nor female XX mice had significant correlations (*p*’s = 0.96, 0.33). *Tlr7* and mean active nose-poke responses over the acquisition period showed similar results, with only XY females having a strong positive correlation (*r* = 0.94, *p* < 0.01; Fig. 8b). *Tlr7* showed another positive correlation with total nose-poke responses, again only in XY females (*r* = 0.94, *p* < 0.05; Fig. 8c).

When we examined all 4 MF1 SCC groups separately, both XX and XY females had a strong positive correlation between *Tlr7* and *Tlr8* (*r* = 0.94, 0.90; *p* < 0.001, *p* < 0.05; respectively; Fig. 8d). XY males also displayed a positive correlation (*r* = 0.89, *p* < 0.05), but XX males only showed a trend (*r* = 0.77, *p* = 0.07). Comparing *Tlr7* mRNA to active nose-poke responses, only XX males had a positive correlation (*r* = 0.89, *p* < 0.05, Fig. 8e). Total nose-poke responses had similar results, with XX males showing a positive correlation with gene expression (*r* = 0.89, *p* < 0.02; Fig. 8f). Behavioral correlations for *Tlr8* showed similar results to *Tlr7* correlation, except MF1 XX male correlations were not significant (Supplemental Table 3).

## Discussion

### No Differences in Vulnerability to Cocaine in B6 mice

Here we found that B6 mice, the strain most widely used in biomedical research, did not display sex differences in vulnerability to cocaine self-administration as assessed by acquisition (FR1) or motivation (PR). This pattern held across all experimental groups, including gonad-intact, gonadectomized, and E2-treated mice. These findings contrast with our previous findings in intact FCG mice derived from the B6 line, as well as decades of research in rats, where females consistently show greater vulnerability during acquisition of cocaine self-administration than males (Lynch et al. 2002; Becker and Koob 2016). Studies in rodents further identify estradiol as a key mechanism driving this enhanced vulnerability, with gonadectomy reducing vulnerability in females but not males, and estradiol restoring this vulnerability in females while generally having little effect in males (Lynch et al. 2002; Becker and Koob 2016; but see Bagley et al. 2019). It is possible that a ceiling effect, reflected by rapid and near-maximal rates of acquisition (78–92%), contributed to the lack of group differences observed here. We did use a slightly steeper dose-escalation paradigm in this study compared to our previous study in FCG B6 mice, which accelerated the acquisition process and may have decreased sensitivity to group differences. However, this explanation alone is unlikely to explain the lack of effects in B6 mice given that group differences were detected in FCG studies conducted under conditions with comparable variability. It is also notable that in rat studies, sex differences can be obscured under conditions that accelerate acquisition and reduce individual variability; however, effects of gonadectomy and estradiol are typically robust and not easily eliminated by acquisition conditions (Lynch et al. 2002; Hu et al. 2004; Caine et al. 2004; Lynch 2006; Jackson et al. 2006; Perry et al. 2013; Becker and Koob 2016; Carroll and Lynch 2016)

Importantly, we also did not observe enhanced vulnerability in gonad-intact female B6 mice. This suggests that female-biased vulnerability is not uniformly expressed across mouse models and may depend on strain background and/or interactions between SCC and gonadal hormones. Indeed, although relatively few studies have examined sex differences in cocaine self-administration in mice, results have been less uniform than those reported in rats. For example, while we and others have replicated greater vulnerability in females than males in studies using CD1 mice (Martini et al. 2014; Castro-Zavala et al. 2021), studies in other lines show either no difference (B6 and B6-derived lines, current study;DePoy et al. 2021; López et al. 2021b; Slosky et al. 2022; Balakrishnan et al. 2024), greater intake in females under conditions in which g/kg dose was not equated between sexes, or greater vulnerability in males, but only under more demanding response schedules (e.g., PR testing; (Griffin et al. 2007; but see López et al. 2021a). This latter finding is notable given that we have also reported higher motivation in showing the slowest acquisition and lowest percent group acquisition (intact FCG B6 XY males, Martini et al., 2020), indicating that acquisition and motivation may dissociate in mice and reflect distinct aspects of cocaine vulnerability, rather than a single underlying vulnerability construct as is often assumed in rats. The idea that genetic background is a key determinant of sex differences in cocaine self-administration in mice is also supported by a recent study in genetically diverse mouse populations showing that sex effects differed across strains and behavioral phases (Dickson et al. 2025).

Consistent with this variability, prior studies suggest that sex and hormone effects in B6 mice are often not observed under baseline conditions and instead emerge under specific genetic or environmental perturbations. For example, Wickens et al. (2021) and Birmingham et al. (2023) found sex differences in cocaine self-administration after knockdown of the protein that interacted with C-kinase-1, Pick1, in the prefrontal cortex, whereas no sex differences were apparent in WT controls receiving GFP. Similarly, DePoy et al. (2021) found that gonadectomy reduced cocaine self-administration in neuronal PAS domain protein 2 (*Npas2)* knockout females, but had no effect in wild-type controls. Together, these findings suggest that in B6 mice, sex differences in cocaine vulnerability may be difficult to detect under baseline conditions and instead emerge in the presence of genetic or environmental manipulations.

### SCC as a Driver of Cocaine Vulnerability

In contrast to the absence of sex and hormonal effects in WT B6 mice, results from the FCG model revealed a different pattern when gonadal hormones were removed. In gonadectomized FCG B6 mice, vulnerability was driven by SCC, with XY mice showing accelerated acquisition and greater responding than XX mice. This pattern also differs from our previous findings in gonad-intact FCG B6 mice, in which vulnerability was greater in females than males and reflected both gonadal sex and SCC effects (XX > XY; Martini et al., 2020). Together, these findings indicate that the pattern of cocaine vulnerability depends on both SCC and gonadal status, with removal of gonadal hormones shifting vulnerability from a female-biased to an SCC-driven phenotype.

The impact of estradiol treatment also appears to differ between FCG B6 and WT B6 mice. In our previous study in gonadectomized FCG B6 mice, estradiol treatment significantly reduced acquisition rates and intake, but only in XY males (Le et al., 2023). Although we used the same estradiol implants in the present study, we did not replicate these effects in WT B6 mice. As such, this difference is unlikely to reflect methodological factors and instead raises the possibility that the impact of gonadal hormones on cocaine vulnerability depends on SCC, a factor isolated in the FCG model but not varied in wild-type mice.

In contrast to the robust SCC effects observed in the B6 FCG line, no effects of SCC were observed in gonadectomized MF1 FCG mice. In fact, apart from modest male–female differences in active responses during acquisition, there were no significant effects of sex or SCC within the MF1 line. Direct comparisons across strains confirmed higher intake and responding in B6 than MF1, driven primarily by XY mice. Together, these findings indicate that SCC effects are not uniformly expressed across FCG models and instead depend on genetic background. One interpretation is that SCC-related differences are modified by strain-specific factors, consistent with prior reports of substantial strain variation in cocaine self-administration (Thomsen and Caine 2006; Dickson et al. 2015, 2016; Leonardo et al. 2023). Alternatively, these effects may be influenced by the translocation of X-linked genes to the Y chromosome in the B6, but not MF1, FCG line (Panten et al. 2024), raising the possibility that the observed SCC effects reflect, at least in part, line-specific genetic features rather than SCC alone.

### Mechanistic Insights into SCC Effects

Sequencing the Y-chromosome in B6 and XY FCG b6 mice revealed a missense SNP in *Zfy2*, a Y-linked zinc finger protein critical for spermatogenesis (Holmlund et al. 2023), but of unknown neural function. Structural modeling predicted that this mutation may render the protein nonfunctional (Lavorando et al. 2024), though its impact was difficult to assess given that the variant was in a region predicted to be disordered. A mutation like this could have arisen as a by-product of backcrossing the Y-chromosome from the original FCG (in the MF1 strain) into B6. The primary source of *Zfy2* expression was the testes, but there was also mRNA present in the embryonic brain (National Center for Biotechnology Information 2025). While *Zfy2* has not been studied in the context of addiction, its X-linked homolog *Zfx* has been implicated in neurodevelopmental disorders (Shepherdson et al. 2024). Although speculative, subtle Y-linked variants such as this SNP could influence neural or behavioral phenotypes. However, the functional relevance of this variant remains unclear and requires further validation. While these findings point to potential Y-linked genetic variation, they do not exclude the possibility that developmental differences in hormone exposure also contribute to the observed phenotypes.

Only one report has examined AGD in FCG B6 mice (Itoh et al., 2015). In that study the predicted sex difference was noted (M > F) and the XX and XY mice of the same gonadal sex were not different. However, comparisons to normal B6 mice were not made. In the FCG, the *Sry* transgene presented a potential caveat (De Vries et al. 2002). The *Sry* transgene was inserted into chromosome 3 with approximately 12 repeats (Itoh et al. 2015). The transgene may have also disrupted the function of nearby genes such as latexin (*Lxn)* and peptidylprolyl isomerase D (*Ppid*; (Itoh et al. 2015), both of which have been linked to behavioral phenotypes (Jin et al. 2006; Bourke et al. 2013; Gunduz-Cinar et al. 2019). The FCG B6 line also differs in Y chromosome origin: WT B6 carry the B6 Y-chromosome, whereas the FCG Y-chromosome originates from the a129/SvEv-Gpilc background (Itoh et al. 2015). These features could play a part in modiying the amount or timing of androgen production by the embryonic testes.

We observed that FCG B6 mice in both sexes had shorter AGD than their normal B6 counterparts, which suggested lower neonatal androgen exposure in FCG mice during the critical period of anogenital formation. AGD is a well-established biomarker of androgen action during perinatal development (Gillette et al. 2025), with embryonic and postnatal males exposed to higher testosterone than females due to the neonatal testes (Palanza et al. 1995). Moreover, studies have shown that male littermates may have influenced female AGD via hormonal diffusion in utero (Vandenbergh and Huggett 1995; Palanza et al. 1995). In mice, differentiation of AG tissues occurs between embryonic day 16 and 10 days after birth (Yamada et al. 2003) when androgen receptors are abundant in genital tubercles of both sexes (Agras et al. 2006). The smaller AGD we observed in FCG litters may have indicated reduced or mis-timed androgen or androgen receptor signaling during critical periods of sexual differentiation. Such differences in early hormone exposure would have influenced the brain and behavioral development as well, providing one novel and important mechanism through which SCC interacted with gonadal factors to shape cocaine-related behaviors. We did not measure AGD in wild-type MF1 mice as they were not presently available in the United States. However, without comparable measurements in MF1 mice, it remains unclear whether these developmental differences generalize across FCG lines.

Finally, in FCG B6 mice, but not in MF1, XY mice of gonadal sexes expressed higher levels of *Tlr7* and *Tlr8* than XX mice in the NAc. We examined *Tlr7* and *Tlr8* because they were two of the 9 X-linked genes duplicated and translocated to the Y-chromosome (Panten et al. 2024). In B6 FCG mice, *Tlr7* and *8* mRNA in NAc were positively correlated, but only in XY females. *Tlr7* expression was also positively associated with active nose-pokes responses in this group, suggesting a relationship with cocaine seeking/taking behavior. This association was not explained by cocaine intake, as *Tlr7* mRNA levels were not significantly associated with cocaine intake. This association was also stronger in XY females than XY males, suggesting a potential interaction between SCC and developmental steroid exposure, which is higher in gonadal males than gonadal females. In contrast, this pattern was not observed in MF1 FCG mice, where only XX males showed a positive correlation between nose-poke responses and *Tlr7* mRNA levels. This divergence across strains is consistent with the possibility that the X-to-Y translocation in the B6 FCG line contributes to the observed associations. Together, these findings suggest that toll-like receptor signaling may be associated with cocaine-related behaviors.

Toll-like receptors are broadly expressed in neurons, astrocytes, oligodendrocytes, and microglia (Ma et al. 2007; Crews et al. 2017; Michaelis et al. 2019; Seizer et al. 2022), and a growing body of work has linked them to drug-related behaviors. For example, TLR2/4 were elevated in brains of alcohol-exposed humans and mice (Crews et al. 2013), behavioral responses to alcohol were reduced in *Tlr2* knockdown mice (Blednov et al. 2017), and TLR4 antagonists blocked cocaine-cued reinstatement after extinction (Brown et al., 2023). *Tlr7* mRNA levels were likewise elevated after binge drinking in mice, and agonists of TLR7 increased voluntary alcohol consumption (Allard et al. 2024). Moreover, a TLR7/8 vaccine reduced fentanyl’s analgesic effects in mice (Powers et al. 2023). Our findings extended this literature through correlations between *Tlr7/8* expression and cocaine self-administration measures, specifically in XY female mice. These results highlight immune signaling pathways as a potential mechanistic link between the immune system and cocaine vulnerability

## Conclusions

We found that gonadal status did not affect vulnerability to cocaine self-administration in wild-type B6 mice. Using two strains of mice in the FCG model, we identified strain differences in the interactions between SCC and gonadal sex. At the mechanistic level, our data suggest potential roles for immune signaling (via *Tlr7/8* expression), early androgen exposure (via AGD), and a Y-chromosome SNP in *Zfy2* in shaping cocaine vulnerability. The duplication of X-linked genes, now overexpressed in XY mice, could have contributed to the strain differences reported here. The developmental effects of the *Sry* transgene on androgen signaling are novel findings with potentially large impacts on previous and future studies using the FCG. The uncertain significance of the *Zfy2* SNP highlights the possibility that these results reflect strain-specific artifacts of B6 SCC-driven mechanisms. However, the association of toll-like receptor genes with cocaine-related behaviors may represent a novel and important pathway for future investigation. These results underscore both the promise and the limitations of the FCG approach: while it reveals candidate biological pathways linking SCC to drug-related behaviors, careful validation in additional models will be required to establish their functional relevance.

## Supplementary Material

Supplementary Files

This is a list of supplementary files associated with this preprint. Click to download.

• SupplementalFig1.png

• SupplementalFig2.png

• SupplementalFig3.png

• SupplementalFig4.png

• SupplementalFig5.png

• SupplementalFig6.png

• SupplementalFig7.png

• SupplementalFig8.png

• SupplementalTables.docx

## Data Availability

The data generated for this study are available at the University of Virginia. Requests for raw data can be directed to the corresponding author.
